# Postoperative complications after gastrointestinal pediatric surgical procedures: outcomes and socio-demographic risk factors

**DOI:** 10.1186/s12887-022-03418-8

**Published:** 2022-06-22

**Authors:** Robert Brock, Angel Chu, Shengjie Lu, Mary Elizabeth Brindle, Ranjani Somayaji

**Affiliations:** 1 Department of Pediatric and Adolescent Medicine, Faculty of Medicine, University Hospital Cologne, University of Cologne, Kerpener Strasse 62, 50937 Cologne, Germany; 2 Department of Medicine, University of Calgary, Calgary, AB Canada; 3 Cumming School of Medicine, University of Calgary, Calgary, AB Canada; 4 Department of Surgery, University of Calgary, Calgary, AB Canada; 5 Department of Microbiology, Immunology, and Infectious Diseases, University of Calgary, Calgary, AB Canada; 6 Department of Community Health Sciences, University of Calgary, Calgary, Canada

**Keywords:** Pediatric gastrointestinal surgery, Postoperative complications, Surgical disparities, Gender, Income, Race

## Abstract

**Background:**

Several socio-demographic characteristics are associated with complications following certain pediatric surgical procedures. In this comprehensive study, we sought to determine socio-demographic risk factors and resource utilization of children with complications after common pediatric surgical procedures.

**Methods:**

We performed a population-based cohort study utilizing the 2016 Healthcare Cost and Use Project Kids’ Inpatient Database (KID) to identify and characterize pediatric patients (age 0–21 years) in the United States with common inpatient pediatric gastrointestinal surgical procedures: appendectomy, cholecystectomy, colonic resection, pyloromyotomy and small bowel resection. Multivariable logistic regression modeling was used to identify socio-demographic predictors of postoperative complications. Length of stay and hospitalization costs for patients with and without postoperative complications were compared.

**Results:**

A total of 66,157 pediatric surgical hospitalizations were identified. Of these patients, 2,009 had postoperative complications. Male sex, young age, African American and Native American race and treatment in a rural hospital were associated with significantly greater odds of postoperative complications. Mean length of stay was 4.58 days greater and mean total costs were $11,151 (US dollars) higher in the complication cohort compared with patients without complications.

**Conclusions:**

Postoperative complications following inpatient pediatric gastrointestinal surgery were linked to elevated healthcare-related expenditure. The identified socio-demographic risk factors should be considered in the risk stratification before pediatric surgical procedures. Targeted interventions are required to reduce preventable complications and surgical disparities.

## Introduction

Surgery can be a high-risk, resource-intensive therapy and postoperative complications contribute significantly to disease burden and healthcare expenditure in countries around the world [1]. Postoperative complications in pediatric patients are distinct from typical complications seen in adults due to the challenges of operating on smaller anatomical structures and specific age-related pathophysiologic processes [2]. Therefore, focused research efforts are needed to evaluate underlying risk factors of postoperative complications in the unique population of pediatric surgical patients.

Previous studies indicate that there are substantial differences in odds of postoperative complications depending on socio-demographic variables in pediatric surgical patients [3–10]. Socio-economically disadvantaged groups and rural populations are likely to experience worse surgical outcomes [11]. Such inequalities are often referred to as *surgical disparities* [11]. In the literature documenting surgical disparities in adults, both patient and provider factors have been identified and are strongly interconnected [12]. Among the recognized socio-demographic patient factors are race, ethnicity, sex and socio-economic status, while provider factors include implicit or unconscious biases, differences in clinical decision-making, and years of training [11]. In pediatric studies of tonsillectomy, female sex and greater household income were associated with a decreased rate of post-tonsillectomy hemorrhage [3] whereas respiratory complications were greater among African-American patients [4]. In the US, African American and Hispanic children were more likely to experience a complication and had longer hospital stays than Caucasian patients after undergoing an appendectomy for acute appendicitis [5]. Insurance status [6–8], hospital location (urban vs. rural) [9], and geographic region [10] are other factors that have demonstrated associations with postoperative outcomes.

A detailed understanding of the interplay of specific factors contributing to postoperative complications is crucial for targeted preventive approaches. However, this interplay remains poorly elucidated for pediatric surgical patients. Most previous studies have had a small sample size or have had a narrowed focus of contributing factors that may lead to complications. Thus, we conducted a comprehensive population-based cohort study to evaluate the role of socio-demographic and center factors on postoperative complications and healthcare costs in inpatient pediatric gastrointestinal surgical patients. We hypothesized that both patient and center characteristics are significantly associated with post-surgical outcomes in this setting.

## Methods

### Study population and data source

In this retrospective cohort study, we used the 2016 Kids’ Inpatient Database (KID) of the Healthcare Cost and Utilization Project (HCUP) to evaluate the characteristics and outcomes of postoperative complications in children undergoing pediatric gastrointestinal surgery. The 2016 KID, released in September 2018, is a nationally representative database of pediatric hospitalizations in the United States and contains data on 3,117,413 pediatric hospital stays from 4,200 hospitals in 47 states [13]. HCUP provides weighting factors based upon census data which can be used to produce national estimates. We used the *International Classification of Diseases – 10th revision – Procedure Coding System* (ICD-10-PCS) to identify pediatric patients aged 0–21 years in the United States undergoing the following common inpatient pediatric gastrointestinal surgical procedures: appendectomy, cholecystectomy, colonic resection, pyloromyotomy and small bowel resection. These procedures were selected to achieve a broad representation of both infant and childhood gastrointestinal disorders across a spectrum of complication risk. Incidences of a wide range of postoperative complications were determined utilizing codes from the ICD-10 section *Complications – surgical procedure.* Analyzed diagnoses included post-procedural endocrine and metabolic complications and disorders (ICD-10 code E89), intraoperative and post-procedural complications and disorders of the nervous system (G97), eye and adnexa (H59), ear and mastoid process (H95), circulatory system (I97), respiratory system (J95), digestive system (K91), musculoskeletal system (M96) and genitourinary system (N99) as well as not elsewhere classified complications of procedures (T81) and other complications of surgical and medical care (T88). In health administrative data, this approach of identifying postoperative complications based on ICD-10 codes has been shown to have high negative predictive value (0.93) relative to prospectively identified surgical complications [14]. ICD-10 diagnostic codes were also used to exclude patients with chronic conditions such as chromosomal aberrations, congenital malformations and metabolic disorders [15]. Patients with necrotizing enterocolitis (NEC; ICD-10 P77) were also excluded from analyses, since the KID does not provide any distinction between preexisting and postoperative NEC [16]. Children were categorized as either having no postoperative complications or having any complication during the admission.

### Statistical analysis

Summaries of the variables were computed for each group prior to significance tests and model construction. Weighted numbers of procedures and complications were used for frequency estimates and unweighted numbers were used in the outcome models in accordance with HCUP data structure and use. Groups were compared with t-tests and proportions were compared with chi-squared testing. Admission trends over the study period were assessed with the Mann-Kendall trend test. Our primary outcome was to identify factors associated with increased risk of postoperative complications.

We constructed a binomial logistic regression model for the occurrence of complications. Procedure type, socio-demographic characteristics, hospital factors and admission features were included as a priori confounders. Socio-demographic characteristics included age, sex (male/female), race (Caucasian/African American/Hispanic/Asian and Pacific Islander/Native American/other), expected primary payer (Medicare/Medicaid/private insurance/self-pay/no charge/other) and household income national quartile based on ZIP code. Patient’s age at admission was categorized into the following four age groups similar to prior methods [17]: neonates (< 1 month), infants (1 month to 2 years), children (2 to 12 years) and adolescents (12 years or older). Hospital factors were hospital region (Northeast/Midwest/South/West), hospital location and teaching status (rural/urban non-teaching/urban teaching) and hospital size. Categories of hospital size were assigned by the HCUP as small (1 to 99 beds), medium (100 to 399 beds), and large (≥ 400 beds). Elective admission, weekend admission and admission month were included as admission features. Gestational age and BMI were included as comorbid conditions. Gestational age categories were extremely premature (< 28 weeks, ICD-10 code P07.2), premature (28 to 36 weeks, P07.3), mature (37 to 42 weeks) and post-term (> 42 weeks, P08.2). BMI was categorized into less than 5th (Z68.51), 5th to 84th (Z68.52), 85th to 94th (Z68.53) and greater than or equal to 95th (Z68.54) percentile for age. Intestinal perforation status (K35.2 and K35.3) was also included in the outcome models. We conducted a subgroup analysis for the outcomes of persons who underwent appendectomy as it was the most common procedure either in isolation or in combination with other surgical procedures.

The secondary outcomes of the study were length of stay (LOS) in days and hospitalization costs in US dollars. Hospitalization costs were computed with the KID Cost-to-Charge Ratio Files. Multivariable linear regression models were constructed to determine the LOS and costs for persons with and without complications. The models were adjusted for the patient and hospital characteristics as above. A second set of models was constructed for procedure-specific results. *A priori* selected covariates with missing values less than 10% were incorporated into the final models. A *p* value less than 0.05 was considered statistically significant for all tests. Analyses were conducted with R 3.6.1 (R Foundation for Statistical Computing, Vienna, Austria).

## Results

A total of 48,022 unweighted corresponding to 66,157 weighted inpatient pediatric gastrointestinal surgical admissions were analyzed. Baseline characteristics for patients with and without postoperative complications are detailed in Table 1. The mean age at admission was 12.1 (SD 5.8) years. There were greater proportions of males (54.7%), of non-Caucasian race (53.8%), on Medicaid (51.0%), in the first (lowest) household income national quartile (31.0%), admitted in the South region (35.2%), and in urban teaching centers (73.5%) at large hospitals (62.9%). Most admissions were non-elective (93.0%) and occurred on a weekday (74.7%).

**Table 1:** Baseline characteristics of inpatient pediatric gastrointestinal surgical patients in the United States in 2016

Variable	Total (*n* = 66,157)	No Complication (*n* = 64,148)	Complication (*n* = 2,009)
Procedure			
Appendectomy	50,947 (77.0)	49,290 (96.7)	1,657 (3.3)
Cholecystectomy	9,796 (14.8)	9,561 (97.6)	235 (2.4)
Colonic Resection	333 (0.5)	297 (89.2)	36 (10.8)
Pyloromyotomy	4,574 (6.9)	4,546 (99.4)	28 (0.6)
Small Bowel Resection	254 (0.4)	228 (89.8)	26 (10.2)
Combination of 2 Procedures	--	--	--
Combination of 3 Procedures	--	--	--
Age in Years mean (SD)	12.2 (5.8)	12.1 (5.8)	12.1 (5.6)
Age Group			
< 1 month	1,694 (2.6)	1,669 (98.5)	25 (1.5)
1–24 months	3,544 (5.4)	3,486 (98.4)	58 (1.6)
2–12 years	27,239 (41.2)	26,312 (96.6)	928 (3.4)
>12 years	33,679 (50.9)	32,681 (97.0)	998 (3.0)
Sex			
Male	36,172 (54.7)	34,896 (96.5)	1,276 (3.5)
Female	29,980 (45.3)	29,249 (97.6)	731 (2.4)
Race			
White	28,619 (46.3)	27,758 (97.0)	860 (3.0)
Black	4,794 (7.7)	4,606 (96.1)	188 (3.9)
Hispanic	22,807 (36.9)	22,189 (97.3)	619 (2.7)
Asian/Pacific Islander	1,729 (2.8)	1,679 (97.1)	50 (2.9)
Native American	588 (1.0)	554 (94.3)	34 (5.7)
Other	3,338 (5.4)	3,225 (96.6)	113 (3.4)
Insurance Status			
Medicare	161 (0.2)	--	--
Medicaid	33,729 (51.0)	32,740 (97.1)	989 (2.9)
Private Insurance	26,916 (40.7)	26,092 (96.9)	824 (3.1)
Self-pay	3,209 (4.9)	3,105 (96.8)	104 (3.2)
No Charge	149 (0.2)	--	--
Other	1,916 (2.9)	1,844 (96.2)	72 (3.8)
Median Income for ZIP Code			
First Quartile	20,238 (31.0)	19,628 (97.0)	610 (3.0)
Second Quartile	16,243 (24.9)	15,706 (96.7)	536 (3.3)
Third Quartile	15,829 (24.2)	15,339 (96.9)	490 (3.1)
Fourth Quartile	13,003 (19.9)	12,655 (97.3)	348 (2.7)
Geographic Region of Hospital			
Northeast	11,245 (17.0)	10,965 (97.5)	280 (2.5)
Midwest	10,568 (16.0)	10,175 (96.3)	393 (3.7)
South	23,308 (35.2)	22,525 (96.6)	784 (3.4)
West	21,036 (31.8)	20,484 (97.4)	552 (2.6)
Hospital Location and Teaching Status			
Rural	4,265 (6.4)	4,086 (95.8)	179 (4.2)
Urban Nonteaching	13,236 (20.0)	12,813 (96.8)	423 (3.2)
Urban teaching	48,656 (73.5)	47,249 (97.1)	1,406 (2.9)
Hospital Size			
Small (1 to 99 beds)	8,706 (13.2)	8,410 (96.6)	296 (3.4)
Medium (100 to 399 beds)	15,818 (23.9)	15,361 (97.1)	457 (2.9)
Large (≥ 400 beds)	41,633 (62.9)	40,377 (97.0)	1,256 (3.0)
Admission Modality			
Non-elective	61,336 (93.0)	59,532 (97.1)	1,804 (2.9)
Elective	4,635 (7.0)	4,433 (95.6)	202 (4.4)
Admission Day			
Weekday	49,401 (74.7)	47,884 (96.9)	1,517 (3.1)
Weekend	16,756 (25.3)	16,264 (97.1)	492 (2.9)
Admission Season			
Winter	16,473 (24.9)	15,982 (97.0)	491 (3.0)
Spring	16,645 (25.2)	16,135 (96.9)	510 (3.1)
Summer	17,437 (26.4)	16,890 (96.9)	547 (3.1)
Fall	15,599 (23.6)	15,138 (97.0)	460 (3.0)
Perforation			
No	42,618 (64.4)	41,868 (98.2)	750 (1.8)
Yes	23,539 (35.6)	22,281 (94.7)	1,258 (5.3)
Gestational Age			
Extremely Immature < 28 Weeks	18 (0.0)	--	--
Immature 28–37 Weeks	64 (0.1)	--	--
Post-term > 42 Weeks	--	--	--
Body Mass Index			
< 5th Percentile for Age	62 (0.1)	--	--
5th − 85th Percentile for Age	174 (0.3)	163 (93.7)	11 (6.3)
85th − 95th Percentile for Age	159 (0.2)	--	--
≥ 95th Percentile for Age	778 (1.2)	748 (96.1)	30 (3.9)
Not available	64,982 (98.2)	63,022 (97.0)	1,961 (3.0)

Column 1 represents column/block based %s; Column 2 and 3 contain row based frequencies and %s. Frequencies that are < 11 and related frequencies were not reported based on HCUP policy

Of these, 51,168 underwent an appendectomy, 9,959 had a cholecystectomy, 4,575 had a pyloromyotomy, 382 had a colonic resection and 331 had a small bowel resection. The complication rates were 3.3% for appendectomy, 2.5% for cholecystectomy, 0.6% for pyloromyotomy, 11.2% for colonic resection and 12.1% for small bowel resection (*p* < 0.001). 65,904 patients underwent one procedure, 248 patients two different procedures and 5 patients three different procedures in the same admission. The complication rate for undergoing one procedure was 3.0%, for two procedures 10.1% and for three procedures 25.1%. A total of 2,009 patients (3.0%) had at least one postoperative complication. Of those, 1,918 had one, 84 had two and 7 had three complications. The most common complications were post-procedural disorders of the digestive system (n = 1,492). Other complications affected the respiratory system (n = 175), circulatory system (n = 40), genitourinary system (n = 39), musculoskeletal system (n = 4) and nervous system (n = 1).

The results of our multivariable model for examining the factors associated with postoperative complications are presented in Table 2. Female sex (OR 0.67, 95% CI 0.60–0.76) and receiving treatment in urban teaching hospitals (OR 0.74, 95% CI 0.60–0.92) were associated with significantly reduced odds of postoperative complications. In contrast, being of African American (OR 1.38, 95% CI 1.13–1.69), Native American (OR 1.71, 95% CI 1.08–2.71) and other (OR 1.30, 95% CI 1.03–1.65) race was associated with a significantly elevated complication risk when compared with Caucasian race. Other significantly associated factors with increased risk of complications were age less than one month (OR 3.71, 95% CI 1.57–8.77) and between one month and two years (OR 2.11, 95% CI 1.32–3.35) as well as having an elective admission (OR 1.51, 95% CI 1.24–1.84).

**Table 2:** Multivariable logistic regression model to evaluate factors associated with postoperative complications

Variable	All procedures		Appendectomy	
	**OR**	**95% CI**	**OR**	**95% CI**
Procedure				
Small bowel resection	Ref	…		
Appendectomy	0.17	0.10–0.29	Ref	…
Cholecystectomy	0.31	0.17–0.54		
Colonic resection	0.82	0.41–1.65		
Pyloromyotomy	0.02	0.01–0.05		
Combination of two procedures	0.8	0.36–1.76	4.75	2.40–9.38
Combination of three procedures	4.22	0.42–42.95	25.71	2.73–242.38
Age group				
>12 years	Ref	…	Ref	…
< 1 month	3.71	1.57–8.77	17.03	7.70–37.68
1–24 months	2.11	1.32–3.35	1.77	0.99–3.17
2–12 years	0.92	0.82–1.05	0.88	0.77–1.00
Sex				
Male	Ref	…	Ref	…
Female	0.67	0.60–0.76	0.7	0.62–0.80
Race				
Caucasian	Ref	…	Ref	…
African American	1.38	1.13–1.69	1.36	1.08–1.73
Hispanic	0.97	0.84–1.12	0.98	0.84–1.14
Asian and Pacific Islander	1.08	0.76–1.52	1.09	0.76–1.57
Native American	1.71	1.08–2.71	1.43	0.82–2.50
Other	1.3	1.03–1.65	1.42	1.11–1.83
Insurance Status				
Medicare	Ref	…	Ref	…
Medicaid	0.51	0.23–1.13	0.58	0.23–1.47
Private Insurance	0.55	0.25–1.20	0.62	0.24–1.57
Self-pay	0.58	0.25–1.31	0.65	0.25–1.72
No Charge	0.89	0.27–2.95	1	0.25–1.72
Other	0.69	0.30–1.60	0.79	0.30–2.13
Median Income for ZIP code				
First Quartile	Ref	…	Ref	…
Second Quartile	1.09	0.94–1.26	1.16	0.99–1.37
Third Quartile	1.08	0.93–1.27	1.09	0.92–1.29
Fourth Quartile	0.91	0.76–1.10	0.93	0.76–1.13
Geographic Region of Hospital				
Northeast	Ref	…	Ref	…
Midwest	1.22	1.00–1.49	1.16	0.93–1.44
South	1.18	0.99–1.41	1.21	1.00–1.47
West	1.01	0.84–1.21	1	0.82–1.21
Hospital Location and Teaching Status				
Rural	Ref	…	Ref	…
Urban Non-teaching	0.94	0.74–1.18	0.96	0.75–1.24
Urban Teaching	0.74	0.60–0.92	0.74	0.58–0.94
Hospital Size				
Small (1 to 99 beds)	Ref	…	Ref	…
Medium (100 to 399 beds)	0.89	0.73–1.07	0.93	0.76–1.14
Large (≥ 400 beds)	0.9	0.77–1.06	0.9	0.76–1.08
Admission Modality				
Non-elective	Ref	…	Ref	…
Elective	1.51	1.24–1.84	1.3	1.00–1.69
Admission Day				
Weekday	Ref	…	Ref	…
Weekend	0.97	0.85–1.10	0.94	0.82–1.08
Admission Month				
Month	1.00	0.99–1.02	1.00	0.98–1.02
Intestinal Perforation				
No	Ref	…	Ref	…
Yes	4.12	3.57–4.75	4.27	3.69–4.94

*CI* Confidence Interval, *OR *Odds Ratio, *Ref *Reference Group

In the multivariable model for those who underwent appendectomy procedures, female sex (OR 0.70, 95% CI 0.62–0.80) and treatment in urban teaching hospitals (OR 0.74, 95% CI 0.58–0.94) were similarly associated with a significant reduction of the postoperative complication odds in this cohort. Being of African American (OR 1.36, 95% CI 1.08–1.73) or other race (OR 1.42, 95% CI 1.11–1.83), age less than one month (OR 17.03, 95% CI 7.70–37.68), having an intestinal perforation (OR 4.27, 95% CI 3.69–4.94) and having one (OR 4.75, 95% CI 2.40–9.38) or two (OR 25.71, 95% CI 2.73–242.38) more procedures had significantly greater odds of postoperative complications.

**Fig. 1:**
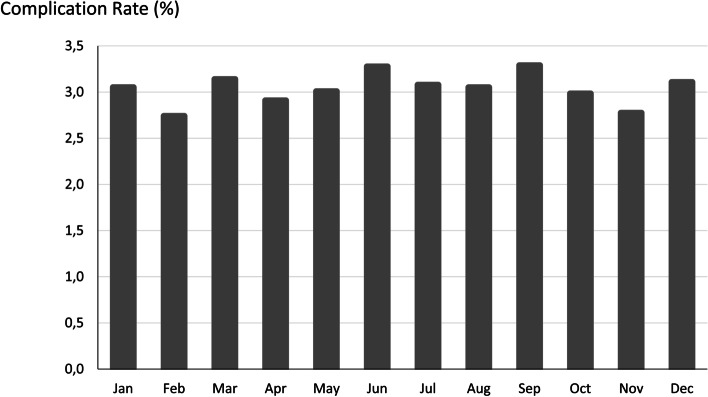
Postoperative complication rate over time in total cohort

Postoperative complication rates were also compared between household income national quartiles for patient ZIP code, geographic regions and between months in separate tests. Complication rates differed significantly among income quartiles for patient ZIP code (*p* = 0.02) and were lowest in patients from fourth (highest) quartiles. Analysis by hospital region showed significant regional differences (*p* < 0.001) with greater case frequencies in the South and Midwest compared with the Northeast and West of the United States. No significant time trend was present in pediatric surgical admissions with or without complications (Fig. 1).

For our secondary outcomes, the mean LOS was 8.30 (SD = 10.01) days in patients with complications and 2.86 (SD = 3.73) days in patients without complications. In the multivariable model, the LOS was significantly greater in the cohort with complications by 4.58 days (95% CI 4.05–5.10). Total hospitalization costs in the presence of at least one complication amounted to an average of $23,674 (SD = 31,116), while the mean costs in the complication-free control group were $10,835 (SD = 13,285). Procedure-specific LOS and costs are presented in Table 3. The adjusted multivariable model revealed a significant difference of $11,151 (95% CI 9,470–12,832) in costs between the two groups. Procedure-specific models are presented in Table 4. The strongest effects of postoperative complications on LOS and costs were found in small bowel resection and colonic resection. Since there were only 15 deaths in the total cohort related to pediatric gastrointestinal surgical procedures, no regression models for mortality were constructed.

**Table 3:** Length of stay and hospitalization costs stratified by procedure type and complication

Procedure		Length of stay		Hospitalization costs	
		**mean**	**(SD)**	**mean**	**(SD)**
All procedures	No complication	2.86	(3.73)	10,835	(13,285)
	Complication	8.30	(10.01)	23,674	(31,117)
Appendectomy	No complication	2.77	(3.17)	10,522	(8,085)
	Complication	7.85	(7.41)	21,231	(24,413)
Cholecystectomy	No complication	3.15	(3.83)	12,598	(24,436)
	Complication	9.66	(19.65)	33,436	(52,209)
Colonic resection	No complication	9.43	(16.35)	32,719	(47,642)
	Complication	17.71	(10.04)	60,431	(50,716)
Pyloromyotomy	No complication	2.42	(3.70)	7,865	(12,551)
	Complication	4.85	(3.62)	14,585	(12,470)
Small bowel resection	No complication	10.82	(17.63)	34,714	(50,135)
	Complication	23.03	(41.15)	64,371	(81,317)

**Table 4:** Multivariable model of length of stay and hospitalization costs by procedure type

Procedure	Length of stay	Hospitalization costs
	**Coefficient (95% CI)**	**Coefficient (95% CI)**
All procedures		
No complication	Ref	Ref
Complication	**4.58 (4.05–5.10)**	**11,151 (9,470–12,832)**
Appendectomy		
No complication	Ref	Ref
Complication	**4.25 (3.81–4.68)**	**9,281 (7,777–10,784)**
Cholecystectomy		
No complication	Ref	Ref
Complication	**5.48 (2.69–8.27)**	**16,722 (10,128–23,316)**
Colonic resection		
No complication	Ref	Ref
Complication	**7.73 (2.84–12.62)**	**34,653 (11,592–57,715)**
Pyloromyotomy		
No complication	Ref	Ref
Complication	0.62 (-1.80–3.03)	2,875 (-5,478–11,228)
Small bowel resection		
No complication	Ref	Ref
Complication	**16.57 (0.18–32.97)**	**37,417 (6,952–67,882)**

## Discussion

Our study of 66,157 admissions representing a national estimate of children undergoing selected gastrointestinal surgical procedures is one of the largest cohorts of pediatric surgical hospitalizations. Our data indicated that infants, males, persons of racial minorities and those treated in rural hospitals had greater odds of experiencing complications following pediatric gastrointestinal surgery. Accordingly, the presence of complications was associated with longer hospitalization stays and higher costs.

We pooled procedures with distinct age-related frequency peaks. Controlling for procedure type in the models enabled the identification of young age as a factor for increased risk of complications after pediatric gastrointestinal procedures. This finding is similar to others that have reported age-related differences in postoperative complication rates in children undergoing adenotonsillectomy or appendectomy [18–20]. We identified that female sex was associated with reduced complication risk across a spectrum of pediatric gastrointestinal surgeries similar to another study of tonsillectomies [3]. While a large body of medical literature addresses sex inequalities in adults, underlying mechanisms in pediatric surgery are unknown. It is possible that differences in postoperative risk especially in the youngest age groups may relate to biological differences in sex but there may also be gendered differences on the part of parents and caregivers that are worthy of further study.

Race is the most commonly investigated patient factor in this research area and racial disparities in outcomes have been demonstrated in a variety of pediatric surgical procedures [4, 5, 21, 22]. Racial health inequity has been linked to inadequate care processes, such as choice of procedure and specialty referral, and may be reinforced by structural racism both within communities and within the healthcare system [23, 24], as well as other socio-cultural factors that are challenging to measure. Accordingly, our data showed increased complication rates among African Americans, Native Americans and patients classified as *other race* compared with Caucasian children. In the few available studies including Native American children, no increased likelihood of postoperative complications was observed [5, 22] whereas we did identify an increased risk suggestive of disparities in surgical access and care. For Hispanic children, the existing evidence on postoperative complication risk is conflicting [4, 5, 8, 22]. Our results support the observations from a recent large cross-sectional study, which did not find an elevated risk for Hispanic children [4] and they did encompass 37% of the cohort.

Consistent with existing evidence, we identified that the hospital location and teaching status were associated with post-surgical complication rates [9]. Our analyses further identified that elective admission was associated with increased odds of complications and may relate to higher complexity of elective procedures compared with typical nonelective procedures; as elective admissions only comprised 7% of the cohort, the accuracy of this result is not clear.

When we examined socio-economic factors, consistent with earlier studies, we noted that there was a trend to lower postoperative complication rates in the highest income groups [3, 25, 26], but this was not significant in the adjusted model. One reason may be the interplay between income and other socio-demographic variables such as race or other unknown confounders [12]. More recent studies have identified primary payer status and hospital region as predictors of postoperative complications [6–8, 10]. Although we did note differences in these factors relative to complications, they were not significant in our multivariable models. Regarding admission features, prior studies have identified weekend admission as a risk factor for complications after pediatric surgery [27, 28] although this was not the case in our cohort. In accordance with previous findings, admission month and hospital size had no effect on complication frequency [29, 30].

Despite this being one of the largest cohorts to evaluate factors of postoperative complications, there are some limitations to consider. As the KID database is an administrative database, there is potential for information bias including misclassification and missingness. However, as the database is rigorous on data accuracy, and we utilized a similar approach to identifying surgery and complications to prior HCUP studies to maintain consistency and ensure inclusion of relevant admissions. As the unit of measure was an admission, it is possible that a patient was discharged before a complication occurred and readmissions were not recorded as linked to the index hospitalization. However, as our focus was on the risk factors and healthcare utilization, a proportion of the cohort having repeated admissions would not have largely affected the results. For missingness, there was minimal to no missingness in the cohort data in the covariates incorporated into the outcome models. BMI and gestational age, potential relevant patient characteristics, were not included in the models due to missingness. As the diagnosis and procedure information is limited to the admission unit of data, the timing and causality of complications to a surgical procedure cannot be definitively determined, but this would have affected the cohort non-selectively and thus likely did not skew the results significantly. Severity of complications was not specifically assessed based on data granularity but encouragingly, less than 4% of the cohort had multiple surgical procedures. Although we did not include all surgical procedures, we focused on the group of surgical procedures with the greatest volume [31], and also thought it would focus the evaluation and maintain our ability to be sufficiently powered for the analyses. We used postoperative complications as an indicator of poor surgical outcome which has some challenges given the lack of agreement in the definition of complications and the grading of the severity. However, we attempted to be comprehensive and our evaluation of associated factors being consistent with earlier studies lends credence to our findings.

The increased use of healthcare resources in patients with complications from our cohort reinforces the relevance of postoperative complications not only to the patients and their families, but also to the healthcare system. To our knowledge, this study provides the most comprehensive analysis of socio-demographic predictors causing disparities in pediatric gastrointestinal surgical outcomes. There is a crucial need for effective and context-sensitive risk stratification algorithms to reduce preventable postoperative complications in pediatric surgical patients from health and socio-economic perspectives. Despite the known impacts of socio-demographic disparities in surgical outcomes, only a minority of surgeons account for these factors in their practice [32] highlighting the need for enhanced education across the medical spectrum as well as quality improvement and health system post-surgical surveillance initiatives.

## Conclusions

In our large population-based cohort study of children undergoing gastrointestinal surgical procedures, we identified a number of patient and system related factors that were associated with disparate outcomes. Young age, male sex, belonging to racial minorities including African Americans and Native Americans as well as receiving treatment in a rural hospital were associated with increased risk of postoperative complications. Only with greater education regarding social determinants of health of medical professionals, healthcare allocation reforms, and optimizing postoperative care pathways can we hope to mitigate outcome disparities in children undergoing surgery.

## Data Availability

The datasets generated and analyzed during the current study are not publicly available due to restrictions but are available from the corresponding author Robert Brock on reasonable request and with permission of the HCUP KIDS’ Inpatient Database (KID), 2016. https://www.hcup-us.ahrq.gov/kidoverview.jsp.

## Abbreviations

BMI: body mass index
HCUP: Healthcare Cost and Utilization Project
ICD-10: International Classification of Diseases – 10th revision
KID: Kids’ Inpatient Database
LOS: length of stay
NEC: necrotizing enterocolitis
